# Histopathological Evaluation of Gastric Mucosal Atrophy for Predicting Gastric Cancer Risk: Problems and Solutions

**DOI:** 10.3390/diagnostics13152478

**Published:** 2023-07-26

**Authors:** Maria A. Livzan, Sergei I. Mozgovoi, Olga V. Gaus, Anna G. Shimanskaya, Alexei V. Kononov

**Affiliations:** 1 Department of Internal Medicine and Gastroenterology, Omsk Sate Medical University, 644099 Omsk, Russia; gaus_olga@bk.ru; 2 Department of Pathological Anatomy, Omsk Sate Medical University, 644099 Omsk, Russia

**Keywords:** chronic atrophic gastritis, *Helicobacter pylori*, autoimmune gastritis, intestinal metaplasia, OLGA/OLGIM, gastric cancer

## Abstract

Patients suffering from chronic gastritis and developing gastric mucosa atrophy are at increased risk of the development of gastric cancer. The diagnosis of chronic atrophic gastritis (CAG) is a complex procedure involving a detailed history taking, a thorough physical examination and the use of laboratory and instrumental diagnostic methods among which the endoscopy of the upper digestive tract is the cornerstone because it allows the assessment of the topography of gastritis and identification of erosions and areas of intestinal metaplasia with the use of NBI endoscopy. However, the diagnosis of CAG requires morphological examination of the gastric mucosa. So, in addition to assessing macroscopic changes in the gastric mucosa, it is necessary to take biopsy specimens in accordance with the protocols for their morphological and immunohistochemical examination. In the absence of specific diagnostic stigmas of CAG, close cooperation between a clinician, endoscopist and pathologist is necessary. The article presents systematized data on the histopathological assessment of the gastric mucosa atrophy to predict the risk of gastric cancer.

## 1. Introduction

Chronic atrophic gastritis (CAG) is the final stage of the inflammatory process, the key characteristic of which is the decrease of gastric glands substitute with fibrous tissue or metaplastic epithelium. The histopathological cascade of gastric carcinogenesis, also called as Correa’s cascade, after the pathologist who first described it in 1975, is a step-by-step process with unchanged gastric mucosa in the beginning followed by chronic superficial gastritis, chronic atrophic gastritis, intestinal metaplasia and dysplasia stages, and cancer in the end [1]. These characteristics describe structural changes in the gastric mucosa at all stages of gastric carcinogenesis and highlight the importance of CAG identification for cancer prevention.

There are two main methodological approaches for the diagnosis of CAG. The first one suggests serological testing using markers of gastric function (pepsinogen I, pepsinogen I/pepsinogen II ratio, additional stimulated and basal gastrin-17), non-invasive testing for *Helicobacter pylori* (*H. pylori*) infection and, if required, endoscopic examination of gastrobiopsy specimens for histopathological verification of atrophy in case of identified atrophy stigmas in the patient. The second approach involves the invasive biopsy procedure from the start for histological analysis of biopsy specimens collected during esophagogastroduodenoscopy.

The purpose of the review is to systematize the data from published studies on the histopathological assessment of gastric mucosal atrophy to predict the risk of gastric cancer.

A systematic search for articles was conducted in the PubMed/MEDLINE, Embase, and Google Scholar databases with the use of the following keywords and their combinations: “chronic atrophic gastritis”, “atrophy of the gastric mucosa”, “histopathological assessment of atrophy”, “intestinal metaplasia of the gastric mucosa”, “risk of gastric cancer”. The selection criterion was full-text articles including original studies, systematic reviews, and meta-analyses published in English up to June 2023. The authors independently reviewed and analyzed the articles. After applying the selection criterion, a total of 90 references were included in this article.

The clinician has to do the following activities during the diagnostic process: to identify the cohort of patients who need to be examined for atrophy detection; to be in close interaction with an endoscopist and pathologist; to conduct a clinical interpretation of the examination results and define a treatment strategy for the patient. Here are the key features of each of the stages of the diagnostic process.

## 2. Who Should Be Examined? Or in Other Words—Who Is at Risk for Chronic Atrophic Gastritis

It is extremely important to identify a number of risk factors for the development of atrophic gastritis and gastric cancer which should be considered by the clinician when making a decision about the active detection of atrophic changes in the gastric mucosa and the need for individual screening. A family history of gastric cancer seems to be one of the strongest risk factors [2,3]. Three case-control studies (in Japan, Poland and South Korea) involving 1024 patients with gastric cancer showed that the odds ratio (OR) for gastric adenocarcinoma in the immediate family of patients with gastric adenocarcinoma ranged from 2.3 to 3.5 [4,5,6]. The findings of another population-based case-control study in the United States revealed that the risk of developing gastric adenocarcinoma was 5–12.1 times higher in individuals having two or more family members with gastric adenocarcinoma [7]. A cohort study in Sweden, Denmark and Finland reported an increased risk of developing gastric adenocarcinoma among monozygotic and dizygotic twins with gastric adenocarcinoma by 9.9 and 6.6 times, respectively [8].

There is evidence that older age is a risk factor for the development of atrophic gastritis and adenocarcinoma of the stomach. Various studies report different age groups (45, 50 or 75 year olds) being at risk [9,10,11]. However, three large studies have shown that >45 year olds have an OR of 1.92 to 3.1 for having the progression from atrophic gastritis to adenocarcinoma [9,12,13]. Therefore, most studies suggest 45 years of age as the cut-off age for screening endoscopic examination [2].

Structural changes in the gastric mucosa are not associated with the symptoms of dyspepsia and their severity, which means that the clinician does not have reliable clinical symptoms allowing them to suspect the atrophy of the gastric mucosa at the stage of questioning and examination [14]. At the same time, with autoimmune genesis of inflammatory changes in the gastric mucosa, the clinical picture can reveal certain clinical and laboratory signs that allow the patient to be included in the cohort of people at high risk of developing gastric mucosa atrophy: female gender, comorbidity with other autoimmune diseases, signs of vitamin B12 and iron deficiency (hematological and neurological manifestations) [15,16].

Thus, the cohort of persons with a high probability of detecting CAG are patients with a combination of dyspepsia syndrome, signs of cyanocobalamin deficiency, anemia and/or other anxiety symptoms, who have a family history of gastric cancer, and those at the age of 45 and older, regardless of other factors.

Additionally, to determine the individual risk, it is necessary to take into account the etiological factor in the development of chronic gastritis.

## 3. The Etiological Factor of Gastritis and the Atrophy Risk

The two most significant etiological factors of CAG are *H. pylori* infection and autoimmune inflammation, with the dominant infectious factor [17,18].

*H. pylori* is a gram-negative, curved or S-shaped microaerophilic bacterium with high motility due to a unipolar bundle of coated flagella [19]. *H. pylori* is thought to have been acquired by modern humans in Africa at least ~100,000 years ago, possibly being transmissed from an unknown animal [20]. The most ancient phylogeographic population of *H. pylori* is hpAfrica2, mainly found in South Africa. Other important, widespread, and recent populations include hpAfrica1, hpNEAfrica, hpEurope, hpEastAsia, hpAsia2, and hpSahul [21,22]. An important step in the evolution of *H. pylori* from the ancestral population of hpAfrica2 to populations that spread around the world was the acquisition of the cag pathogenicity island (cagPAI), which encodes the components of the Cag T4SS protein complex [23,24], surrounding the bacterial cell membrane and facilitating the delivery of various effector molecules into host cells after attachment.

*H. pylori* is well adapted to colonize a unique ecological niche in the deep near-wall mucus layer of the antral mucosa. Several mechanisms, including motility, urease production, adhesion, and others, are important for *H. pylori* colonization [25,26,27].

*H. pylori* colonization of the gastric mucosa induces a proinflammatory response involving various immune cells in the mucosal layer resulting in chronic active gastritis [28]. The severity of inflammation varies greatly in individuals depending on bacterial, host and environmental factors [29,30]. The most important determinant of the pro-inflammatory activity of the *H. pylori* strain is its functional cagPAI [31,32]. Expression of additional host interaction factors, such as a set of adhesins that promotes strong binding to epithelial cells depends on the variable composition of host receptors [33].

About 80% of people with *H. pylori* infection are asymptomatic, but all infected individuals develop gastritis with unpredictable and potentially severe individual outcomes [34,35]. Based on the data from a meta-analysis by Adamu M.A. et al., the risk of developing CAG in patients with *H. pylori* infection was 5.0 (95% CI, 3.1–8.3) times higher than in uninfected patients, in whom the rate of progression of chronic gastritis to CAG was <1% per year [36].

About 90% of gastric cancers are reported to be associated with *H. pylori* infection [37]. The lifetime risk of gastric cancer is 1–5% in individuals with *H. pylori* infection, depending on ethnicity and environmental factors [34,38]. Some populations are at increased risk of gastric cancer after being infected with *H. pylori*, likely due to genetic, socioeconomic factors, and dietary preferences, such as increased consumption of salty or pickled foods among East Asian populations [20,39].

Between 2014 and 2020, the global prevalence of *H. pylori* infection among adults decreased from 50–55% to 43% [34,40], which is mainly due to improved socio-economic status, living standards and hygiene conditions, as well as with the introduction of effective schemes of eradication therapy [20,40,41]. The higher prevalence of *H. pylori* infection among the elderly compared with children is due to the fact that the introduction of *H. pylori* infection mostly occur (90%) in childhood and *H. pylori* persists throughout life, rather than the elderly are at higher risk of being infected [20].

It is generally accepted that eradication therapy is the main measure for the prevention of gastric cancer [42,43]. At the same time, it is important to emphasize that the optimal time for eradication is when gastric atrophy has not been developed yet, since the patients who have already developed intestinal metaplasia or atrophy before the eradication therapy are still at increased risk of gastric cancer even after the elimination of *H. pylori* infection [42,44,45]. A year later after successful eradication therapy, 28.2% of patients have clear inflammatory changes in the gastric mucosa [46]. Shibata W. et al. demonstrated that the restoration of changes in the gastric mucosa after successful eradication of *H. pylori* may take up to 10 years in some patients [47]. The persistence of chronic inflammatory infiltrate is associated with an increase in the activity of lipid peroxidation enzymes and the production of reactive oxygen species. Under conditions of “oxidative stress”, irreversible damage to cell DNA occurs. The cells with the damaged DNA, being accumulated over time, become a “starting point” for the development of gastric cancer in the future. Therefore, it is extremely important to identify a group of patients with ex-*H. pylori* gastritis who are at high risk for developing gastric cancer for timely cancer prevention [48,49]. Amid formed atrophy of the gastric mucosa after the elimination of *H. pylori* infection, dysbiosis of the upper digestive tract, including colonization of the stomach by other microorganisms (*Helicobacter* spp., *Proteus mirabilis*, *Citrobacter freundii*, *Klebsiella pneumoniae*, *Enterobacter cloacae*, *Staphylococcus aureus*), which produce nitrosamines with procarcinogenic potential, contributes to continuous inflammation process and preserves the risk of developing gastric cancer [50,51].

Of particular interest is the fact that an increasing number of patients have endoscopic or histological evidence of gastric mucosal atrophy despite no history of eradication [52]. Many of these cases may be due to inadvertent eradication of *H. pylori* by antibacterial drugs prescribed for other diseases [53].

In addition to association with *H. pylori*, CAG may also be primarily of autoimmune nature due to the production of autoantibodies to gastric parietal cells and/or intrinsic Castle factor. The prevalence of autoimmune gastritis (AIG) in the population ranges from 1 to 8% [54]. The risk group of patients with autoimmune inflammation of the gastric mucosa includes women suffering from autoimmune diseases (e.g., type 1 diabetes mellitus, autoimmune thyroiditis), as well as celiac disease [55]. It is noteworthy that the experts of the Maastricht Consensus addressed the problem of the diagnosis of autoimmune gastritis [42]. For example, one of the provisions (WG 2 Diagnostics Statement 6) states that gastric functional serology (pepsinogens I-II and gastrin levels), anti-*H. pylori* antibodies, anti-intrinsic factor and anti-parietal cell auto-antibodies may provide clinically valuable information on the likelihood of gastric mucosal atrophy, including its aetiology (agreement: 98%, grade 1 A).

It should be noted that with primary AIG, the risk of neuroendocrine tumors increases compared with other etiological factors of gastritis, but the risk of gastric adenocarcinoma is lower than with multifocal atrophy of the gastric mucosa due to *H. pylori* infection (involving the mucous membrane of the antrum and body). Epidemiological studies estimate the incidence of gastric adenocarcinoma among patients with autoimmune gastritis as 14.2 cases per 1000 person-years [56]. A study carried out in Sweden reported that the risk of developing gastric cancer in patients with autoimmune gastritis was 7.4 versus 1.4 cases per 1000 patient-years in the general population [57], and a study performed in Finland reported a similar value risk with a standardized incidence rate of 5.0 [58].

## 4. Possibilities of Endoscopic Examination and Sampling of Biopsy Specimens for the Diagnosis of Gastric Mucosa Atrophy

Endoscopic examination plays a key role in the diagnosis of CAG, since the competency of the endoscopist and the adequacy of gastrobiopsy sampling determine the subsequent morphological assessment of the lesion of the gastric mucosa and verification of the diagnosis [59]. The key data for the diagnosis of chronic atrophic gastritis, which can be obtained from the results of endoscopic examination, are the topography of gastritis and the actual identification of atrophy and metaplasia zones [60]. The inflammation of the gastric mucosa is usually considered from the standpoint of a predominant lesion of an organ part: body gastritis, antrum gastritis, pangastritis [61,62]. Clinical interpretation of the topography of inflammation can be as follows. The body gastric mucosa predominantly involved in the inflammatory process may indicate the presence of AIG [63]. In adults, and in cases of introduction of *H. pylori* infection during adolescence, inflammation begins in the antrum, in the so-called “ecological niche” of the *H. pylori* bacterium [20]. Further spread of the *H. pylori* infection in the proximal direction results in the additional involvement of the body of the stomach in the inflammatory process. Pangastritis is formed in this way. Regardless of the etiology, the dominance of gastric lesions is an unfavorable sign in terms of the risk of developing gastric cancer [44].

According to the data published in the literature, the sensitivity and specificity of conventional white light endoscopy for diagnosing gastric mucosal atrophy are 53–59%, and those of high-definition white light endoscopy with magnification are 70–74% [64]. The study by Zhang Q. et al. presents findings of a meta-analysis of the data collected from 1724 patients which indicate that the combined sensitivity and specificity of white light endoscopy for diagnosing early gastric cancer were 48% and 67%, respectively [65]. Moreover, a meta-analysis of 22 studies showed that almost 10% of gastric cancers could potentially be missed during white light endoscopy, mainly it relates to adenocarcinoma of the body of the stomach [66].

To overcome the diagnostic limitations of standard white light endoscopy in detecting premalignant changes in the gastric mucosa, various imaging enhanced endoscopy (IEE) techniques have been developed, including dye chromoscopy, high-resolution imaging, virtual chromoscopy, and artificial intelligence [65,67,68].

It should be noted that any endoscopic method is superior to serological tests in the aspect of its informativeness concerning the detection of atrophy of the gastric mucosa. However, the MAPS II guidelines, with a high level of evidence (94%) and agreement (94%), recommend high-resolution endoscopy with chromoendoscopy rather than high-resolution white light endoscopy for the diagnosis of premalignant and early neoplastic changes in the gastric mucosa [64].

Chromoendoscopy (CE) is an IEE technique that sprays dyes onto the surface of the gastric mucosa to improve visualization of the lesions under study. The use of CE in the screening of malignancies and premalignant changes in the gastric mucosa can increase the detection rate and provide more accurate visualization of the boundaries of the lesion, which helps to differentiate benign or inflammatory changes from suspected precancerous or malignant ones and determine the zones for biopsy [60,69]. CE has a relatively low cost, and can be used in any endoscopy department, but it is procedurally difficult and more time-consuming. CE with acetic acid, methylene blue and indigo carmine are the main chromoendoscopy methods having higher accuracy than high-resolution white light endoscopy for the diagnosis of early gastric cancer (*p* = 0.005) and precancerous changes in the gastric mucosa (*p* = 0.001) [66,70].

Virtual or electronic chromoendoscopy are imaging methods allowing a detailed examination of the gastric mucosa. Their use increases the efficiency of diagnosing precancerous changes and makes it easier for the endoscopist to select areas “suspicious” for intestinal metaplasia or dysplasia for taking gastrobiopsy specimens without any dye techniques. The methods are easy to use and less time-consuming than when using dyes [60,71,72].

One of the virtual chromoendoscopy methods is narrow band imaging (NBI) developed by Olympus (Olympus Medical Systems Co., Ltd., Tokyo, Japan). The principle of NBI endoscopy is based on an optical phenomenon in which the depth of penetration of light into tissues depends on the wavelength. NBI uses narrow spectra of blue light (415 nm) and green light (540 nm) due to light filters installed in the illuminator, which allows you to get a clearer, more detailed image of the gastric mucosa [60]. The sensitivity and specificity of NBI endoscopy for the diagnosis of the gastric mucosa atrophy reach 95 and 98.5%, and for the diagnosis of early gastric cancer, they are 83% and 96%, respectively [64]. However, taking into account its high cost and invasiveness, in most countries NBI endoscopy is not a screening method for CAG and it is used with patients of risk groups only after a serological examination.

There is less evidence to support the use of other virtual chromoendoscopy techniques such as i-Scan digital contrast enhancement and flexible spectral imaging color enhancement (FICE). At present, there is insufficient evidence to recommend routine clinical use of these methods, although theoretically they may have specificity and sensitivity similar to NBI [73,74].

The standard for the study of biopsy specimens is the OLGA-system protocol which involves taking two fragments from the body of the stomach, two fragments from the antrum and one fragment from the incisura angularis of the stomach, and allows assessing the stage of the process and the risk of developing gastric cancer [75]. In a detailed presentation, modern recommendations are indicated as follows: two biopsies from the antrum of the stomach at a distance of 2 cm from the pylorus along the lesser and greater curvatures, one biopsy from the incisura angularis and two biopsies from the body of the stomach at a distance of 8 cm from the rosette of the cardia along the lesser and greater curvatures [76].

However, the sampling of 5 or more fragments of the gastric mucosa, for example, additional ones in case of the areas of the gastric mucosa suspicious of epithelial dysplasia [77,78,79], does not guarantee the quality of the study, since a strict differentiation of the topography of the biopsy is necessary (the antrum and body of the stomach should be typed by a morphologist), or there might be artificial deformation in the process of tissue embedding. This can be avoided by the orientation of the biopsy material. Biopsy specimens are correctly oriented, allowing to obtain sections perpendicular to the surface of the gastric mucosa, including the muscularis mucosae. Oriented material makes it possible to increase the coefficient of interobserver agreement and achieve a higher level of agreement between diagnostic opinions when assessing the gradation of the level of atrophy of the gastric mucosa [80].

**Figure 1:**
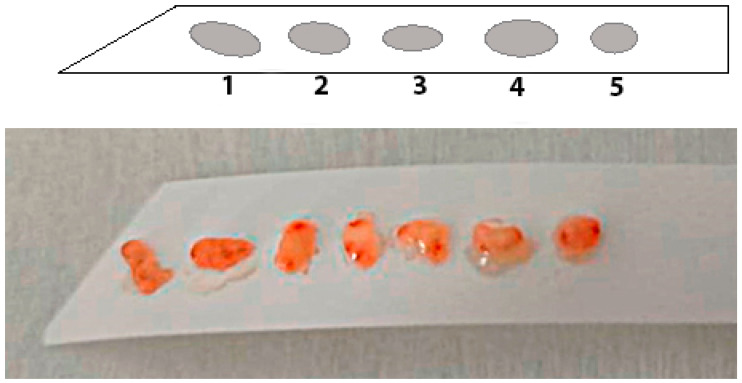
Adhesive orientation strip for biopsy material with tissue fragments located on it. The sequential order of the tissue fragments in the direction from the pointed end allows the marking of the fragments. The photo presents a sequence of 7 oriented tissue fragments.

The use of special adhesive strips can be one of the effective approaches to solve the problem of orientation and fragmentation of biopsies. Orientation using specialized adhesive strips made of cellulose acetate can be carried out by fixing tissue fragments to the strip using manual pressing for 5 s. In this case, the first biopsy specimen is placed at the pointed end of the strip, which provides intuitive recognition of the serial number of the biopsy tissue sample (Figure 1).

After the biopsy specimens have been oriented on the adhesive strip, the biopsy material undergoes a standard processing procedure with fixation in formalin and embedding in paraffin medium. The use of a special adhesive substrate made of cellulose acetate makes it possible to achieve a clear identification of the topography of gastrobiopsy sampling and successful orientation of the biopsy material in most of the cases under study due to the absence of the need to separate the substrate from the biopsy specimens. As a rule, orientation of fragments fails to be done only with a small initial volume of the biopsy material.

## 5. Histological Examination—“Pitfalls” of a Standard Study

In the OLGA (Operative Link for Gastritis Assessment) predictive system, the severity of mucosal atrophy is commonly referred to as the stage of gastritis [62]. There are five stages: stage 0—there is no atrophy, minimal risk of gastric cancer; stages I and II—there is a moderate risk; stages III–IV—there is a high risk (5–6 times higher than in the population). The grade of gastritis is understood as the intensity of infiltration of the gastric mucosa by inflammatory cells: mononuclear and segmented leukocytes together, and not separately, as in the Modified Sydney system [81,82].

The stage of gastritis is a predictive indicator that is used and clinically interpreted much more often than the degree [83]. The degree is usually treated in the same way as the indicator “inflammation” in the Modified Sydney system. The higher grade, the more pronounced the inflammatory cell infiltration, the more cytokines are formed in the gastric lamina propria, the more intense the transformation of specialized cells of the gastric glands into epithelium of intestinal phenotype—intestinal metaplasia—metaplastic atrophy. Therefore, the grade of gastritis is an indirect indicator of the intensity of the patient’s inflammatory response and, therefore, a high degree reflects the acceleration of the development of the carcinogenesis cascade (Correa’s cascade) from chronic inflammation to gastric cancer [84,85].

The assessment of a gastritis stage (severity of atrophy of the glands) is quite subjective. Atrophy is a concept that is not quite accurately identified for a practicing pathologist, which causes insufficient reproducibility of conclusions by different specialists [86]. An additional difficulty for the identification of atrophy is the inflammatory infiltrate, which persists even under the conditions of *H. pylori* eradication and spreading glands. The term “indefinite atrophy” is used to designate such a condition [87,88]. It is assumed that as the inflammation regresses, the judgment about the presence or absence of atrophy will be more objective.

Another thing is intestinal metaplasia, a morphologically striking phenomenon, reproduced by pathologists with a high degree of agreement. The OLGIM (Operative Link on Intestinal Metaplasia Assessment) system has been developed for a predictive assessment of the risk of developing gastric cancer based on the identification of intestinal metaplasia in the biopsy specimen. If inflammation and indefinite atrophy can regress with timely and rational therapy, then intestinal metaplasia has no tendency to reverse development [89].

**Figure 2:**
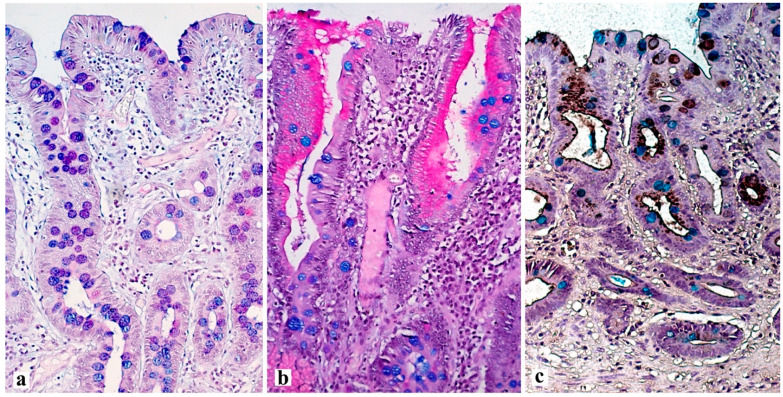
Various types of intestinal metaplasia of the gastric mucosa. (**a**) complete intestinal metaplasia (type I) with a well-defined magenta stained brush border between the goblet cells stained with purple or blue. (**b**) incomplete intestinal metaplasia (type II) with the presence of mucus-producing epithelium between goblet cells, with the production of magenta stained neutral (in this case) or blue stained sialomucins. (**c**) incomplete intestinal metaplasia (type III) with production of sulfomucins by cylindrical cells brownish *black stained* with HID (high iron diamine) stain. (**a**,**b**)—PAS stain with alcian blue (pH = 2.5), (**c**)—high iron diamine with alcian blue (pH = 2.5). ×200.

Intestinal metaplasia is a stigma of atrophy and is defined as the transformation of gastric glandular epithelium into intestinal epithelium. By prevalence, intestinal metaplasia is categorized as limited if the pathological process is located in one anatomical region of the stomach, or as extensive if two regions of the stomach are involved. According to the type of mucins in the lining epithelium, intestinal metaplasia is divided into complete and incomplete. Complete (type I) intestinal metaplasia is similar to the epithelium of the small intestine, and incomplete (type ΙΙ and type ΙΙΙ) intestinal metaplasia is similar to the epithelium of the large intestine (Figure 2).

Relatively recently, the corpus-predominant gastritis index has been included in the diagnostic practice. It is calculated from the totality of microscopic signs of gastritis in the Modified Sydney system. If these signs have higher gradations in the body of the stomach in comparison with the antrum, then the patient has a high risk of developing gastric cancer. The authors believe that this indicator is more accurate than the staging of atrophy/metaplasia according to the OLGA and OLGIM systems of grading [90].

**Figure 3:**
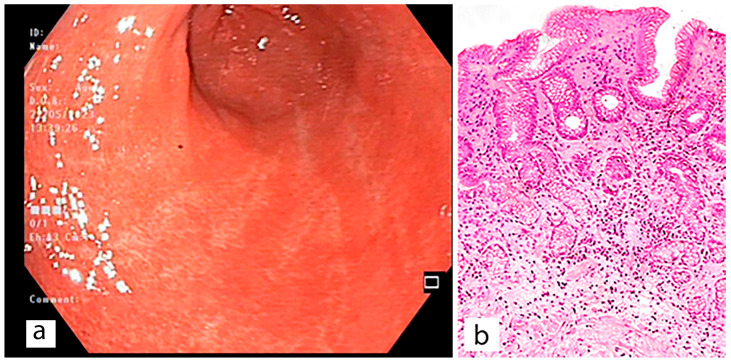
Antrum-gastritis. (**a**) the gastric mucosa in the antrum with foci of atrophy of white color and areas of intact gastric epithelium of red color. (**b**) moderate atrophy of the glands (grade 2). Stained with hematoxylin and eosin (×200).

**Figure 4:**
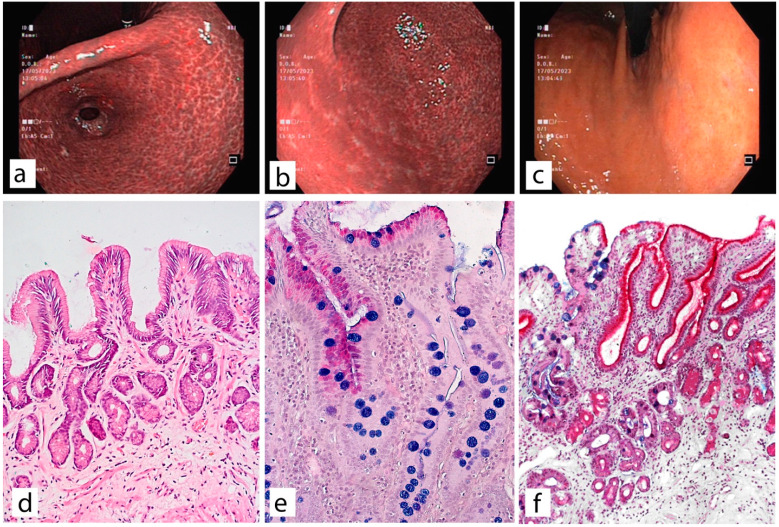
Atrophic gastritis, Kimura-Takemoto O-1. Widespread atrophy and intestinal metaplasia with damage to the antrum, angle and body of the stomach. (**a**) atrophy of the gastric mucosa in the antrum along all walls with a transition to the gastric incisura angularis, examination in the NBI mode. (**b**) the F-line is clearly visible along the greater curvature at the border of the antrum and the body of the stomach. (**c**) examination in white light in retroflexion, atrophy extends along the lesser curvature to the upper third of the body of the stomach. (**d**) biopsy material from the antrum of the stomach, moderate atrophy of the glands. (**e**) biopsy material from the incisura angularis with signs of complete (type I) and incomplete (type II) intestinal metaplasia. (**f**) biopsy material from the body of the stomach with signs of widespread intestinal metaplasia (blue stained goblet cells) and pseudopyloric metaplasia (magenta stained mucus producing cells). (**d**)—staining with hematoxylin and eosin; (**e**,**f**)—PAS stain with alcian blue (pH = 2.5). (**d**,**e**) ×200, (**f**) ×150.

**Figure 5:**
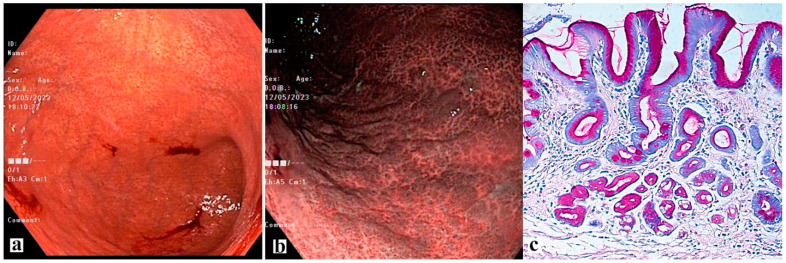
Autoimmune gastritis. The antral gastric mucosa (**a**) is brightly spotty hyperemic along all the walls, foci of the residual glandular mucous membrane are visible, and the vascular pattern is not traced. (**b**) the severity of atrophy is much greater in the body of the stomach. (**c**) typical morphological changes in the mucosa of the body of the stomach. In most fundic glands, chief and parietal cells are replaced by PAS-positive magenta stained mucin-producing cells (pseudopyloric metaplasia), and foci of complete intestinal metaplasia with magenta stained goblet cells are presented. PAS stain with azur-eosin (×200).

Macroscopic and corresponding microscopic changes in the gastric mucosa, reflecting the presence of atrophic gastritis, are shown in Figure 3, Figure 4 and Figure 5.

Immunohistochemical examination in the diagnosis of atrophy has a supporting role. As a rule, the phenomenon of gland decrease is well visualized during routine histopathological examination. Gastrointestinal mucins can be used to better identify the glandular structures, especially in the case of a pronounced inflammatory infiltrate, to assess the level of functional maturity of cells. Of particular importance is the identification of the transformed cell phenotype, the so-called metaplastic atrophy [18,91,92].

Distinguishing its two main types—intestinal and pseudopyloric (pyloric) metaplasia according to modern diagnostic approaches, the emphasis is shifting towards the diagnosis of SPEM (spasmolytic polypeptide-expressing metaplasia) as a special cell line associated with the risk of developing gastric adenocarcinoma rather than the ordinary intestinal metaplasia [76].

Table 1 presents potential markers of metaplastic transformation of the gastric mucosa, ranging from early markers of its restructuring (CDX2) to proteins associated with the formation of a specialized phenotype (TFF2, AQP5, CD44v9, Hep).

**Figure 6:**
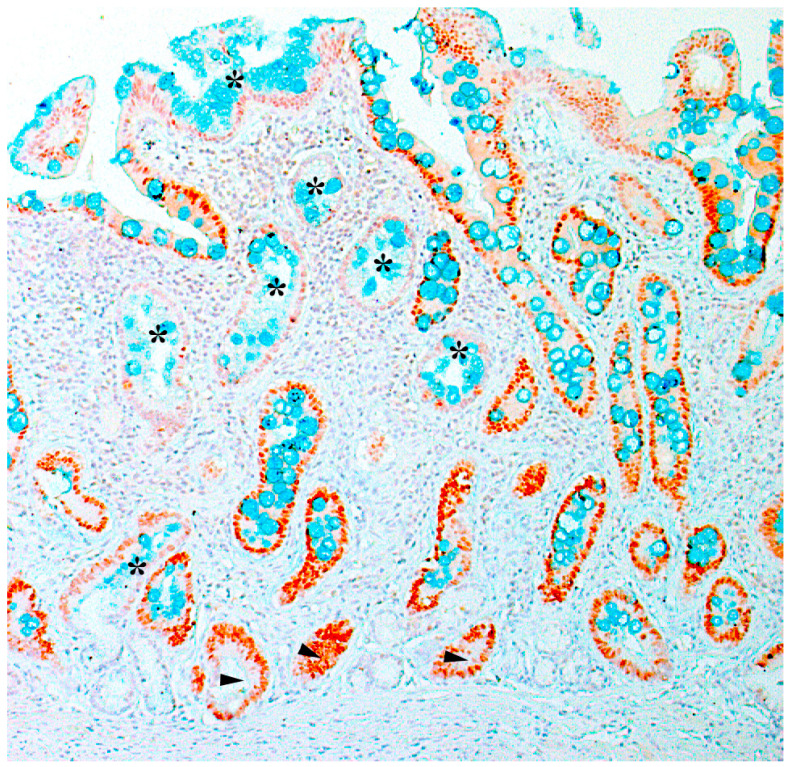
A fragment of the antral gastric mucosa with widespread intestinal metaplasia. Immunohistochemical expression of CDX2 (nuclear brown staining) with additional alcian blue staining (pH = 2.5) of goblet cells (blue stained). Nuclear expression of the marker is present in the metaplastic epithelium and is less pronounced (looked paler) in the foci of incomplete intestinal metaplasia with mucin-producing cylindrical cells (asterisks), and is also present in the residual gastric glands with the absence of goblet cells (arrows). ×200.

Immunohistochemical expression of CDX2 with additional staining of goblet cells with alcian blue is shown in Figure 6.

## 6. Conclusions

The active introduction of OLGA/OLGIM system into the clinical practice allows the assessment of an individual’s risk of developing gastric cancer in a patient with chronic gastritis. The significant problem is the decrease in the informativeness of the histopathological assessment of chronic gastritis due to fragmentation and orientation of the biopsy material, which requires the use of special approaches. An additional opportunity in the study of biopsy specimens of the gastric mucosa is the identification of signs of gastritis specific for autoimmune inflammation and *H. pylori* infection. Modern approaches with the use of immunohistochemical markers in the examination of biopsies of the gastric mucosa play an auxiliary role in the identification of atrophy and intestinal metaplasia, and they may be important in determining the cell phenotype in ambiguous diagnostic cases.

## Figures and Tables

**Table 1:** Possible markers of atrophy and metaplasia of the gastric mucosa.

Marker	Functional Role	Normal Expression	Expression in Pathological Condition	Associated Metaplasia
MUC1	formation of mucosal protective barrier	foveolar epithelium of the body and antrum of the stomach	lack of expression	positive expression in incomplete intestinal metaplasia
MUC2	formation of mucosal protective barrier	goblet cells	goblet cells of metaplastic epithelium	complete intestinal metaplasia
MUC5AC	formation of mucosal protective barrier	foveolar epithelium of the body and antrum of the stomach	lack of expression	pseudopyloric metaplasia positive expression in incomplete intestinal metaplasia
MUC6	formation of mucosal protective barrier	lower part of antral glands and neck cells	similar to TFF2	pseudopyloric and incomplete intestinal metaplasia
CDX2	intestinal transcription factor	absent	cell nuclei in glands, which are transformed to intestinal metaplasia	intestinal metaplasia
Hep	urea metabolism	hepatocytes, small intestinal epithelium	cells of metaplastic glands	incomplete intestinal metaplasia
CD44v9	cell adhesion factor	absent	cytoplasm and membrane of damaged epithelial cells	SPEM
SOX9	transcription factor	neck of the gastric glands of the antrum	basal part of metaplastic glands	intestinal metaplasia, SPEM
TFF2	Formation of mucosal barrier of the stomach	mucocytes of neck of the gastric glands of the body of the stomach, lower part of of antral glands	cytoplasm of mucocytes of metaplastic glands	SPEM
TFF3	formation of mucosal protective barrier	goblet cells	in goblet cells of metaplastic glands	intestinal metaplasia
AQP5	water-channel protein	lower part of antral glands, stem cells	increase expression	pseudopyloric metaplasia, SPEM, intestinal metaplasia

## Data Availability

Not applicable.
